# miR-636 inhibits EMT, cell proliferation and cell cycle of ovarian cancer by directly targeting transcription factor Gli2 involved in Hedgehog pathway

**DOI:** 10.1186/s12935-020-01725-7

**Published:** 2021-01-20

**Authors:** Jiong Ma, Chunxia Zhou, Xuejun Chen

**Affiliations:** Department of Gynecology, The Second Affiliated Hospital of Zhejiang University School of Medicine, No.88 Jiefang Road, Hangzhou, 310009 China

**Keywords:** miR-636, Gli2, Ovarian cancer, Proliferation, Migration

## Abstract

**Background:**

Hedgehog (Hh) signaling pathway, which is essential for cell proliferation and differentiation, is noted to be aberrantly activated in tumor from increasing studies in recent years. MicroRNAs (miRNAs) as an important non-coding RNA in cells have been proven to possess a regulatory role specific to the Hh signaling pathway. Here, in vitro and in vivo cellular/molecular experiments were adopted to clarify the regulatory mechanism linking miR-636 to the Hh signaling pathway in ovarian cancer (OVC).

**Methods:**

Protein–protein interaction analysis was performed to identify the hub gene in the Hh pathway. TargetScan database was used to predict the potential upstream regulators for Gli2. qRT-PCR was performed to test the expression of miR-636, while Western blot was conducted to detect the expression of proteins related to the Hh pathway and epithelial-mesenchymal transition (EMT). For cell functional experiments, HO-8910PM OVC cell line was used. MTT assay and wound healing assay were used to measure the effect of miR-636 on cell proliferation and migration. Flow cytometry was carried out to examine the effect of miR-636 on cell cycle, and Western blot was used to identify the change in expression of Hh and EMT-related proteins. Dual-luciferase reporter gene assay was implemented to detect the targeting relationship between miR-636 and Gli2. Xenotransplantation models were established for in vivo examination.

**Results:**

Gli2 was identified as the hub gene of the Hh pathway and it was validated to be regulated by miR-636 based on the data from TargetScan and GEO databases. In vitro experiments discovered that miR-636 was significantly lowly expressed in OVC cell lines, and overexpressing miR-636 significantly inhibited HO-8910PM cell proliferation, migration and induced cell cycle arrest in G0/G1 phase, while the inhibition of miR-636 caused opposite results. Dual-luciferase reporter gene assay revealed that Gli2 was the target gene of miR-636 in OVC. Besides, overexpressed miR-636 decreased protein expression of Gli2, and affected the expression of proteins related to the Hh signaling pathway and EMT. Rescue experiments verified that overexpression of Gli2 reversed the inhibitory effect of miR-636 on HO-8910PM cell proliferation and migration, and attenuated the blocking effect of miR-636 on cell cycle. The xenotransplantation experiment suggested that miR-636 inhibited cell growth of OVC by decreasing Gli2 expression. Besides, overexpressing Gli2 potentiated the EMT process of OVC cells via decreasing E-cadherin protein expression and increasing Vimentin protein expression, and it reversed the inhibitory effect of miR-636 on OVC cell proliferation in vivo.

**Conclusion:**

miR-636 mediates the activation of the Hh pathway via binding to Gli2, thus inhibiting EMT, suppressing cell proliferation and migration of OVC.

*Trial registration:* The experimental protocol was established, according to the ethical guidelines of the Helsinki Declaration and was approved by the Human Ethics Committee of The Second Affiliated hospital of Zhejiang University School of Medicine (IR2019001235). Written informed consent was obtained from individual or guardian participants.

Ovarian cancer (OVC) belongs to gynecological malignancies, and most OVC cases are diagnosed at advanced stages due to their concealed lesion sites. According to the latest data revealed in *A Cancer Journal for Clinicians*, OVC takes up 2.5% of all malignancies among females1. Given a relatively high mortality which accounts for 5% of all cancer-related deaths, OVC has become the fifth leading cause of cancer-related death in women^2^. OVC can be classified as epithelial OVC, non-epithelial OVC and metastatic OVC, among which epithelial OVC is the most common type in females of all races and nationalities, accounting for 90% of all OVC cases [3]. At present, the effective treatment for OVC is surgery plus chemotherapy, but up to 80% of patients still experience recurrence after chemotherapy [4]. Thus, it is urgent to develop novel molecular targeted treatment for patients with OVC, which requires an in-depth understanding on cell signaling pathways and molecular functions related to tumor occurrence and development. Current studies are mainly devoted to studying cell proliferation and migration of OVC and finding novel molecular targets, so as to improve clinical therapeutic effect and prognosis of patients.

MicroRNAs (miRNAs) refer to a class of single-stranded non-coding RNA molecules with a length of 20–24 nucleotides, and they are widely present in eukaryotes. Besides, miRNAs are highly conservative in evolution, and they are capable of binding to the 3′-untranslated region (3′-UTR) of target genes to induce mRNA degradation or inhibit mRNA translation, which ultimately plays a part in cell differentiation, proliferation and apoptosis [5–7]. Currently, emerging evidence reveals the vital role of miRNAs in regulating tumor initiation and progression. For instance, anti-miR-203 in breast cancer lowers tumor growth and reduces stemness via targeting SOCS3 [8]. miR-454-3p and miR-374b-5p suppress bladder cancer cell migration and invasion with the downstream target as ZEB2 [9]. Moreover, miR-26a and miR-26b in esophageal squamous carcinoma can lead to decrease in cell proliferation, which is realized by deactivation of c-MYC pathway [10].

Hedgehog (Hh) pathway is one of the signaling pathways vital in the embryonic period and plays a role in maintaining the development of tissues and organs normal via regulating cell proliferation, differentiation, epithelial-mesenchymal transition (EMT) and stem cell maintenance. It is reported that abnormal activation of the Hh signaling pathway in adult tissues is associated with the formation, self-renewal and drug-resistance of various tumors [11]. Increasing evidence has showed that EMT in malignant tumors is responsible for enhanced migratory and invasive abilities of tumor cells, and it helps tumor cells develop drug-resistance to conventional treatment [12–14]. Regarding the association between miRNAs and the Hh signaling pathway, large number of studies show that miRNAs are involved in regulation of tumor cell proliferation and migration with the participation of the Hh signaling pathway. miR-214, for example, is found to inhibit SuFu (Hh pathway inhibitor) protein expression in breast cancer to activate the Hh signaling pathway [15]. Additionally, miR-7-5p and miR-506 are noted to play an inhibitory role in bladder cancer and human cervical cancer by regulating the Hh pathway-related transcription factor Gli3 [16, 17].

Given the fact that solid tumors are proven to effectively respond to diverse treatment strategies in a large number of clinical trials [18–20], further identification of the molecular mechanisms underlying tumorigenesis and progression will bring benefit to cancer diagnosis and treatment, and in turn improve prognosis and life quality of patients suffering cancers. In this study, we found that miR-636 exhibited significant low expression in OVC and it was capable of targeting and regulating the transcription factor Gli2 involved in the Hh pathway, yet the molecular mechanism by which miR-636 regulates the Hh pathway in OVC remains elusive. To this end, we investigated the mechanism linking miR-636 to OVC cell proliferation and metastasis by conducting a series of in vitro as well as in vivo experiments, and thus to gain more insight into the pathogenesis of OVC and to provide a new thought for further clinical diagnosis and treatment.

## Materials and methods

### Cell lines and clinical samples

OVC cell lines composed of HO-8910 (3111C0001CCC000280), HO-8910PM (3111C0001CCC000281), CoC1 (3111C0001CCC000368), Caov-3 (3111C0001CCC000339), and Caov-4 (3111C0001CCC000367) were purchased from the cell resource center of Institute of Basic Medical Sciences, Chinese Academy of Medical Sciences. Human normal ovarian cell line HOSEpiC was ordered from the cell bank of Chinese Academy of Sciences (Shanghai, China). All the cell lines were grown in RPMI 1640 medium (Gibco, 11875093) containing 10% fetal bovine serum (FBS; Gibco, 10099141C) in 5% CO_2_ at 37 ℃.

Paired clinical OVC tumor tissue samples (n = 30) and adjacent normal tissue samples (n = 30) were collected from OVC patients admitted to The Second Affiliated Hospital of Zhejiang University School of Medicine, and all of the tissue samples were immediately frozen in liquid nitrogen at -80 ℃ after surgical excision. All the patients received no preoperative chemotherapy or radiotherapy, and signed Informed Consent before participation. This study was approved by the Ethics Committee of The Second Affiliated Hospital of Zhejiang University School of Medicine.

### Bioinformatics analysis

Genes associated with the Hh signaling pathway were searched from Kyoto Encyclopedia of Genes and Genomes database (KEGG, https://www.kegg.jp/kegg/pathway.html). Functional association analysis was performed for the identified Hh-related genes by means of constructing a protein–protein interaction (PPI) network on the STRING database (https://string-db.org/). TargetScan database (http://www.targetscan.org/vert_71/) was consulted to search the potential upstream regulatory miRNAs of Gli2, while Gene Expression Omnibus (GEO) database (https://www.ncbi.nlm.nih.gov/geo/) was consulted to obtain a microarray GSE58517 (5 normal tissue samples and 5 OVC tissue samples) for OVC miRNA expression data. Normal tissue samples were used as control, and differential analysis was performed by using the “limma” package in R, with |logFC|> 2 and *p* value < 0.05 set as critical values.

### Gene overexpression and knockdown

miR-636 mimic, miR-636 inhibitor, agomiR-636 and negative control (mimic NC, inhibitor NC, and agomiR-NC) were all purchased from Shanghai GenePharma Co., Ltd. Short hairpin RNA (shRNA) targeting Gli2 for gene silencing was synthesized by Sangon Biotech Co., Ltd (Shanghai). pEGFP1 vector was used to establish pEGFP1-Gli2 recombinant plasmid for Gli2 overexpression. Transfection was carried out using Lipofectamine®3000 (Invitrogen company, USA) according to the instructions.

### Real-Time fluorescence quantitative PCR (qRT-PCR)

Total RNA was extracted from tissues and cells using Trizol (Invitrogen), and then cDNA was synthesized using the reverse transcription kit (Invitrogen). qRT-PCR was run on the ABI 7900HT Systems (Applied Biosystems, USA) with the miScript SYBR Green PCR Kit (Qiagen, Germany) under the following thermal cycling conditions: pre-denaturation at 95 ℃ for 10 min, followed by 40 cycles of 95 ℃ 2 min, 95 ℃ 5 s and 60 ℃ 30 s. Expression of Smo, Gli2, Snail, Vimentin, Tgfβ, E-cadherin was normalized with GAPDH as an internal reference, and miR-636 expression was normalized with U6 as an internal reference. Relative expression of target genes in control group and test group were compared by 2^−ΔΔCt^ value. All primers used were shown in Additional file 1: Table S1.

### Western blot

After transfection for 48 h, cells in different treatment groups were washed with pre-cooled phosphate buffered saline (PBS; Thermo fisher, USA) for 3 times. Whole cell lysate was then used for cell lysis on ice for 10 min, and the extracted protein samples were quantitated using the BCA protein assay kit (Thermo Fisher Scientific, USA). Subsequently, the protein samples were subjected to sodium dodecyl sulfate–polyacrylamide gel electrophoresis (SDS-PAGE) at 100 V following 10 min of boiling at 95 ℃ with 10 μl of loading buffer, after which the separated proteins were loaded on a nitrocellulose membrane at 100 mA within 120 min. After blocked with 5% bovine serum albumin (BSA) or Tris-Buffered Saline Tween (TBST) for 60 min, the membrane was incubated overnight at 4 °C with primary antibodies, followed by addition of secondary antibody goat anti-rabbit IgG conjugated with horseradish peroxidase (HRP) for hybridization at a room temperature within 120 min. The membrane was transferred to a shaking table for wash with 1 × TBST (Solarbio, Beijing, China) before and after the second incubation, with the wash respectively ran for 3 times. The electrochemiluminescence kit from Solarbio (Beijing, China) was employed to visualize protein bands. All antibodies used were detailed in Additional file 1: Table S2.

### Cell proliferation assay

MTT method was implemented to examine cell viability in proliferation. HO-8910PM cells were seeded into 96-well plates at a density of 5 × 10^3^ cells/well, and each treatment was run in triplicate. After 1 d, 2 d, 3 d, 4 d and 5 d, respectively, sterile MTT solution (Beyotime) was added to the cells per the instructions. The absorbance value at 490 nm was measured by an enzyme-labeled instrument (Molecular Devices, Sunnyvale, CA, USA).

### Wound healing assay

For wound healing assay, HO-8910PM cells (1 × 10^6^) were planted into 6-well plates, and then cell monolayers were wounded with a 200 μl sterile pipette tip when cells grew to 80% in confluence. Isolated cells were removed with mediums, and the cells remained were cultured in fresh mediums for 24 h. Images were photographed at 0 h and 24 h and the wound closure rate was measured.

### Flow cytometry

For cell cycle analysis, the cells after 48 h of transfection) of different groups were collected and digested with 0.25% trypsin. Then, the cells were washed with PBS and re-suspended by 70% ice-cold ethanol (1 ml) for 24 h at 4 °C. Afterwards, the cells were exposed to propidium iodide (PI) and ribonuclease for staining from light for 30 min (4 ℃). Flow cytometry was ran according to the standard procedure and ModFit software was applied to analyze the obtained data.

### Dual-luciferase assay

To verify whether miR-636 can directly target and bind to Gli2 3′UTR, wild-type (WT) and mutant-type (MUT) Gli2 3′UTR segments were separately inserted into psiCHECK luciferase reporter vectors (Sangon Co., LTD, Shanghai, China). Subsequently, the established vectors (psiCHECK-WT/MUT) were transfected into HO-8910PM cells, together with miR-636 mimic or mimic NC. The cells before transfection were incubated for 24 h in a 48-well plate. Finally, luciferase activity of each group was determined by using the luciferase assay kit from Promega (Fitchburg, WI, USA).

### Mouse experiment

A total of 20 male nude mice (6-week-old) from Shanghai SLAC Laboratory Animal Co., Ltd were included in this study for in vivo experiment, and they were all housed under sterile conditions (12/12 h, dark/light; 25 ℃; 60–70% humidity). Specifically, HO-8910PM cells (1 × 10^6^) were firstly inoculated into the abdomen of the mice. When tumor volume reached 70 mm^3^, two groups were set, with the mice in one group (n = 10) injected with agomiR-636 (10 nmol/50 ml) and the mice in the other group (n = 10) injected with agomiR-NC as control. Injection was performed twice a week for consecutive 4 weeks. Tumor volume measurement followed the formula as below: Volume (cm^3^) = (length × width ^2^)/2. After 4 weeks of treatment, the nude mice were euthanized, and the in vivo tumors were isolated, weighed and photographed. Mice care and laboratory procedures were carried out following the ethics committee recommendations for laboratory animals.

### Immunohistochemistry (IHC)

The tumor tissue samples isolated from mice were firstly exposed to 4% paraformaldehyde and placed in a refrigerator with a temperature of 4 ℃. After gradient dehydration with different concentration of ethanol, the samples were treated for transparency with dimethylbenzene and then paraffin-embedded. Immunochemical staining was ran after the tissue blocks were cut into slices [21]. Thereafter, the slices were treated with dimethylbenzene and finally sealed using neutral balsam for slides preparation. All the antibodies used were detailed in Additional file 1: Table S2.

### Statistical analysis

All the data obtained in this study were processed using SPSS 22.0 software. Mean ± standard deviation (SD) was used to express measurement data, while *t* test was used for comparison between two groups. *p < 0.05 was considered poorly statistically significant, **p < 0.01 was considered median statistically significant, while ***p < 0.001 was considered highly statistically significant. All the cell experiments in this study were performed with three technical and biological repeats.

## Results

### Expression of miR-636 and proteins related to Hh pathway and EMT in OVC cell lines

**Fig. 1:**
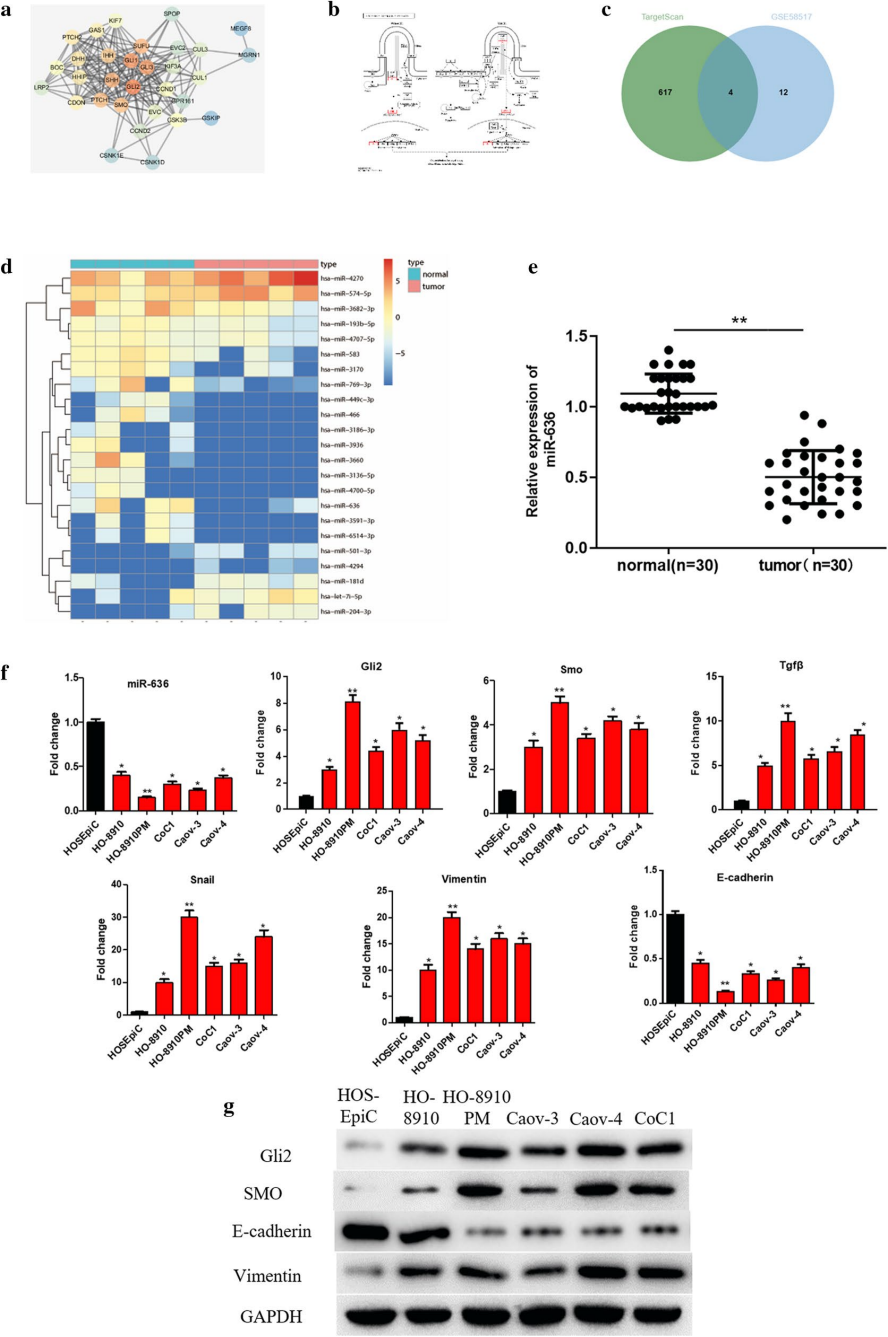
Differential expression of miR-636, Hh and EMT markers in OVC. **a** Association analysis for Hh signaling pathway-related genes (each circle represents a gene and the edges between circles indicate associations); **b** Hh signaling pathway in KEGG database (red represents the location of Gli2 gene in the signaling pathway); **c** The intersection between genes predicted by TargetScan database and down-regulated DEmiRNAs; **d** Heat map shows the DEmiRNAs in GSE58517 dataset; **e** The expression of miR-636 in clinical cancer tissue samples (n = 30) and adjacent normal tissue samples (n = 30); **f** Differential expression of miR-636, Hh and EMT markers in OVC cells and normal ovarian cells detected by qRT-PCR; **g** The protein expression of Hh and EMT markers examined by Western blot; *p < 0.05, **p < 0.01

Several studies found that the Hh signaling pathway is abnormally activated in tumor and affects tumorigenesis and development [22–24], while Gli gene is an important player involved in this pathway. In this study, genes associated with the Hh signaling pathway were firstly searched on the KEGG database **(**Additional file 1: Table S3). At last, 37 genes were obtained and then projected onto the STRING network for functional association analysis (Fig. 1a). It was found that Gli2 was in the center of the network, and our further retrieval of the location of Gli2 in the Hh signaling pathway also revealed that the Gli2 gene was in a center place (Fig. 1b). To learn more about the upstream regulatory mechanism of Gli2, GSE58517 microarray consisting of OVC miRNA expression data was obtained from GEO database. Differential analysis was performed and then 23 differentially expressed miRNAs (DEmiRNAs) were screened (Fig. 1d), among which 16 miRNAs were considerably down-regulated in tumor samples. In the meantime, potential upstream regulatory miRNAs of Gli2 were predicted by TargetScan database, and then taken to make an intersection with the 16 down-regulated DEmiRNAs, contributing to 4 candidate miRNAs (Fig. 1c). As analyzed in GSE58517 dataset (Table 1), miR-636 was found to down-regulate in OVC in the highest degree (logFC = − 5.88). Besides, qRT-PCR analysis based on clinical tissue samples (tumor: n = 30; adjacent normal: n = 30) was performed, showing that miR-636 was notably down-regulated in cancer tissues (Fig. 1e), which implied that miR-636 may play a crucial role in occurrence of OVC.

**Table 1:** Differential expression of the 4 candidate miRNAs in GSE58517 dataset

Symbol	logFC	*P* value
miR-3170	− 5.832962968	0.015331487
miR-466	− 5.30360532	0.026900184
miR-636	− 5.884162127	0.036453732
miR-3591-3p	− 4.465207808	0.040734882

To further explore the role of miR-636 in OVC, we firstly verified the expression of miR-636 as well as the Hh signaling pathway and EMT markers in OVC cell lines. Results of qRT-PCR showed that expression levels of miR-636 and E-cadherin in OVC cells were significantly lower than those in normal ovarian cells, while Hh-related genes (Smo, Tgfβ, Gli2, Snail) and Vimentin were remarkably higher (Fig. 1f). Furthermore, Western blot found that E-cadherin protein expression was evidently lower in OVC cells than that in normal ovarian cells, while Smo, Gli2 and Vimentin were significantly up-regulated (Fig. 1g), which was consistent with the results of qRT-PCR. Taken together, these findings elucidated that the Smo-Gli2-miR-636 axis in OVC may lower tumor growth by suppressing EMT.

### miR-636 inhibits OVC cell proliferation and migration and induces cell cycle arrest in G0/G1 phase

**Fig. 2:**
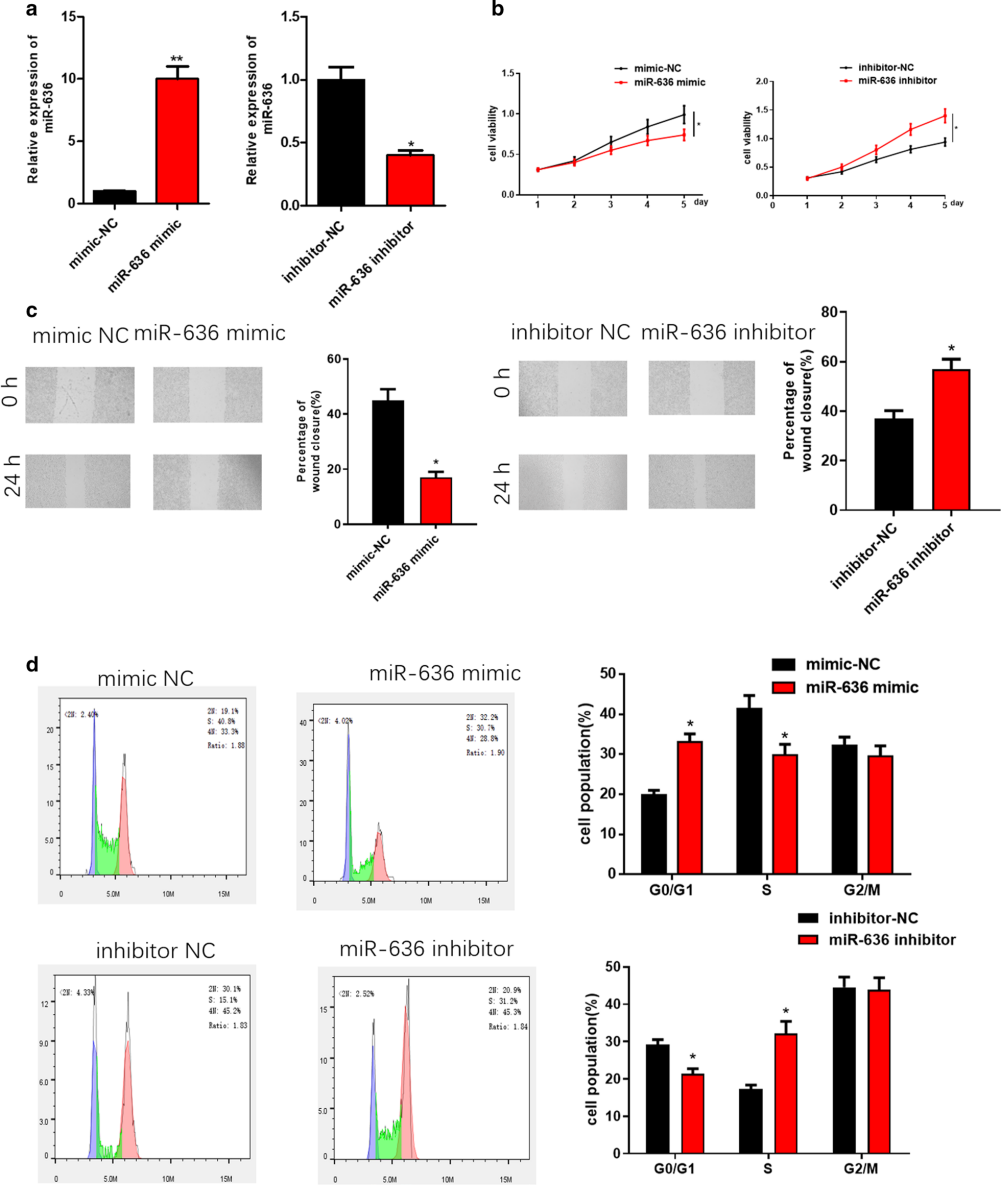
miR-636 inhibits OVC cell proliferation and migration and makes cell cycle arrest in G0/G1 phase. **a** Relative expression of miR-636 in OVC cell line HO-8910PM upon overexpression or inhibition of miR-636; **b** MTT assay, **c** Wound healing assay and **d** flow cytometry showed the effect of miR-636 overexpression/silencing on cell proliferation, migration and cell cycle, respectively; *p < 0.05, ** p < 0.01

To investigate the effect of miR-636 on the growth of OVC cells in vitro, miR-636 mimic and miR-636 inhibitor were transfected into OVC cell line HO-8910PM, respectively. Results of qRT-PCR showed that the expression of miR-636 in HO-8910PM cells was significantly increased and decreased after transfection of miR-636 mimic and miR-636 inhibitor, respectively (Fig. 2a). Subsequently, MTT assay and wound healing assay were implemented to examine the effect of miR-636 on proliferation and migration of HO-8910PM cells, finding that overexpression of miR-636 markedly suppressed OVC cell proliferation and migration in vitro, while inhibition of miR-636 posed an opposite effect (Fig. 2b, c). Since miR-636 could significantly inhibit cell proliferation, the effect of miR-636 on OVC cell cycle was further investigated. Flow cytometry was performed on cells with elevated or reduced miR-636, showing that overexpressed miR-636 blocked HO-8910PM cell cycle in G0/G1 phase, while suppressed miR-636 remarkably decreased the number of HO-8910PM cells in G0/G1 phase (Fig. 2d). Collectively, these findings validated that miR-636 inhibited OVC cell proliferation, migration in vitro and blocked OVC cell cycle in G0/G1 phase by serving as a tumor suppressor.

### miR-636 suppresses the Hh signaling pathway and EMT process by directly targeting Gli2

**Fig. 3:**
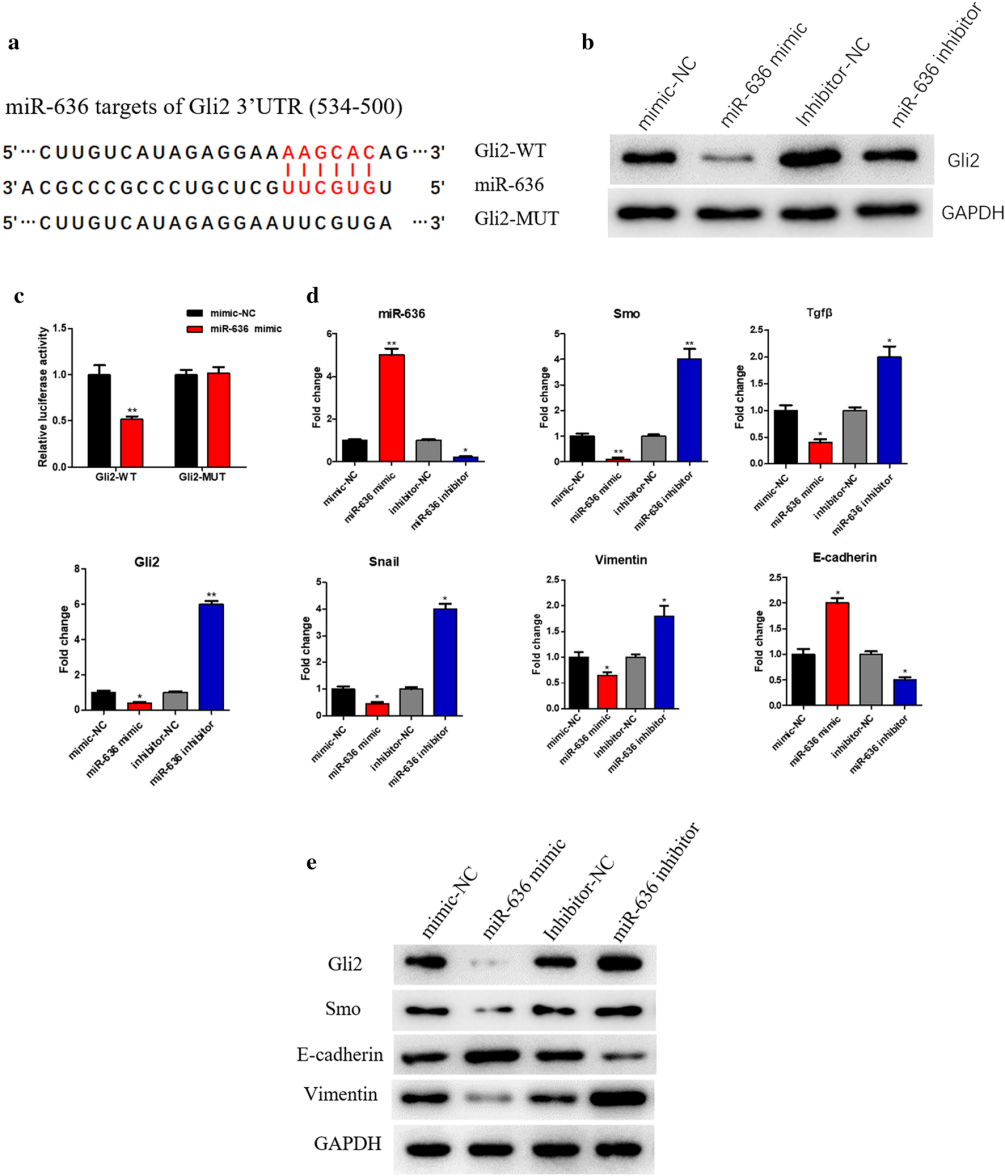
miR-636 directly targets Gli2, inhibits the activation of the Hh signaling pathway and suppresses EMT. **a** The binding sites of miR-636 on Gli2 3′UTR were predicted by TargetScan database; **b** Relative protein expression of Gli2 after miR-636 was overexpressed or inhibited; **c** The targeting relationship between miR-636 and Gli2 was verified by dual-luciferase assay; **d** qRT-PCR was performed to detect the expression of miR-636, Hh and EMT markers in OVC cells with the overexpression or inhibition of miR-636, while **e** Western blot was conducted to examine the protein expression of Hh and EMT markers; *p < 0.05, **p < 0.01

We had predicted that miR-636 might target to regulate Gli2 in OVC as described in *2.1*. To further investigate the molecular mechanism of miR-636 regulating OVC cell cycle, cell proliferation and migration, Western blot and dual-luciferase assays were firstly employed to determine whether miR-636 could directly target Gli2. Based on the TargetScan database, there were potential targeted binding sites of miR-636 on Gli2 3′UTR **(**Fig. 3a). The results of Western blot suggested that overexpressing miR-636 inhibited the protein expression of Gli2, whereas inhibiting miR-636 expression led to the up-regulation of Gli2 (Fig. 3b). Besides, dual-luciferase assay demonstrated that overexpression of miR-636 inhibited the luciferase activity of Gli2-WT, but had no influence on that of Gli2-MUT (Fig. 3c).

Besides, expression of the Hh signaling pathway and EMT markers was examined via qRT-PCR, finding that overexpressed miR-636 in HO-8910PM cells remarkably decreased the expression of Smo, Tgfβ, Gli2, Snail, and Vimentin while increased E-cadherin expression, and opposite results were observed after miR-636 was inhibited (Fig. 3d). Moreover, Western blot indicated that overexpression of miR-636 decreased the expression of Smo, Gli2, Vimentin and increased E-cadherin expression, while opposite results were observed after inhibiting miR-636 (Fig. 3e). To sum up, it was found that miR-636 could directly target to Gli2 to suppress the activation of the Hh signaling pathway and the EMT process, thereby decreasing HO-8910PM cell proliferation and migration.

### Gli2 plays a major role in miR-636-mediated Hh pathway in HO-8910PM cells

**Fig. 4:**
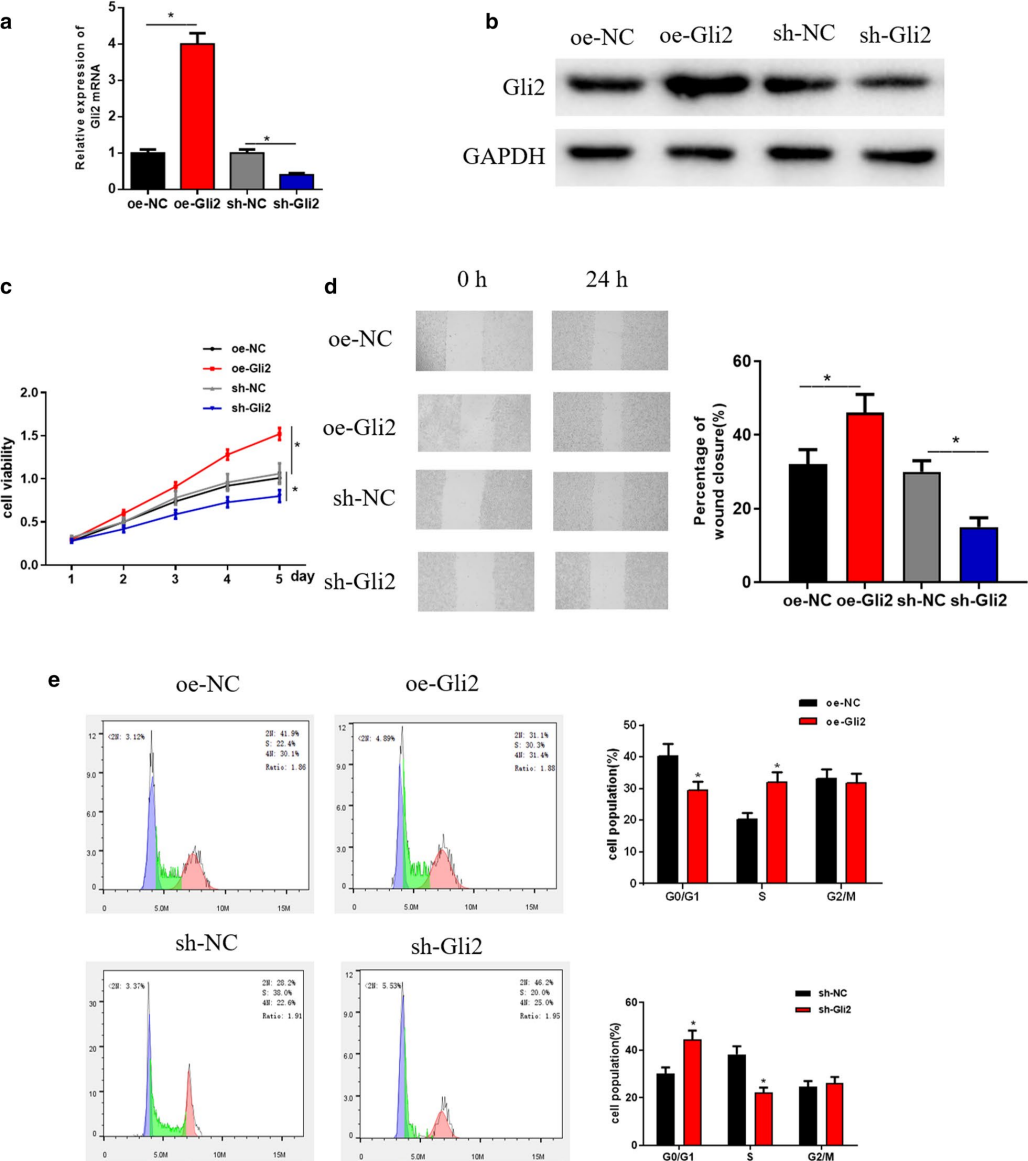
Gli2 plays an important role in cell proliferation, migration and cell cycle of OVC. **a**, **b** Relative level of Gli2 mRNA and protein expression after overexpressing or silencing Gli2 detected by qRT-PCR and Western blot; The effects of Gli2 overexpression or inhibition on cell **c** proliferation, **d** migration and **e** cell cycle were detected by MTT assay, wound healing assay and flow cytometry, respectively; *p < 0.05

Gli2 is a transcription factor with highly conserved C2H2-Zn finger DNA-binding domains [25]. As an effector molecule or a major activating transcription factor in the downstream of the Hh pathway, Gli2 is reported to be a key regulator in various malignancies [26, 27]. In order to verify the effect of Gli2 on OVC cell activities, qRT-PCR and Western blot were firstly conducted to examine Gli2 mRNA and protein expression after overexpressing or silencing Gli2. As the results revealed, Gli2 mRNA expression exhibited an upward trend after oe-Gli2 was applied, while its expression was significantly decreased when sh-Gli2 was transfected (Fig. 4a). Besides, similar trends could be observed in protein expression as judged by Western blot (Fig. 4b). Furthermore, MTT assay was performed to assess the effect of overexpression or silencing of Gli2 on proliferation of HO-8910PM cells, finding that overexpression of Gli2 significantly enhanced proliferation of HO-8910PM cells in vitro, while opposite effect was presented after silencing Gli2 (Fig. 4c). The results of wound healing assay indicated that overexpressing Gli2 significantly enhanced the migratory ability of HO-8910PM cells, while silencing Gli2 noticeably suppressed cell migration (Fig. 4d). Flow cytometry was also performed to detect the cell cycle of HO-8910PM, and it was found that overexpressed Gli2 noticeably decreased the number of HO-8910PM cells in G0/G1 phase, whereas silencing Gli2 made HO-8910PM cell cycle arrest in G0/G1 phase (Fig. 4e). Taken together, these findings elucidated that Gli2 could promote cell proliferation and migration of OVC cells in vitro, and played an important regulatory role in miR-636-mediated Hh signaling pathway in HO-8910PM cells.

### Rescue experiments confirm that miR-636 inhibits cell proliferation, migration and EMT by regulating Gli2

**Fig. 5:**
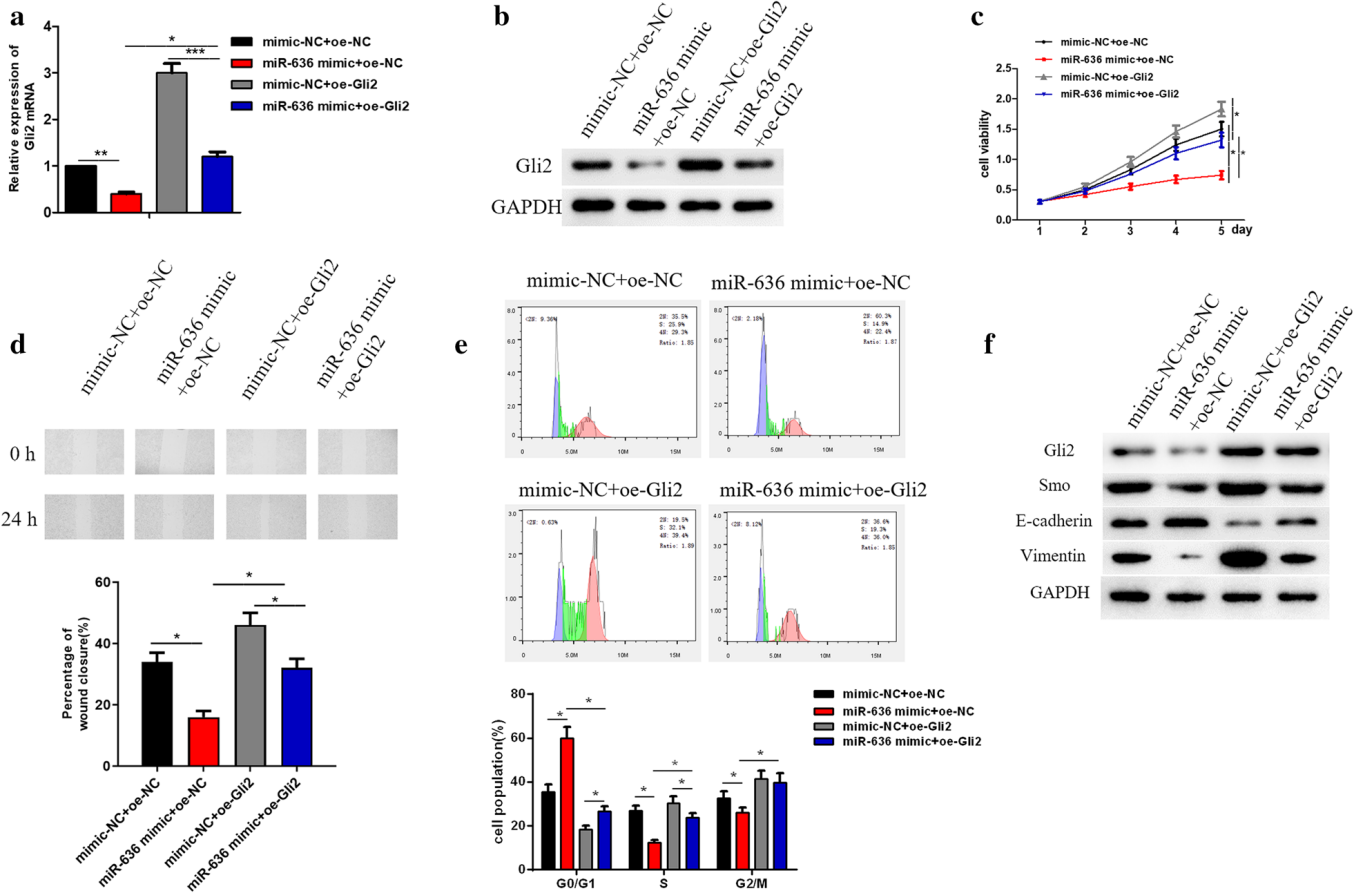
Rescue experiments confirm that miR-636 inhibits cell proliferation and EMT by regulating Gli2. **a**, **b** Relative levels of Gli2 mRNA and protein expression were detected by qRT-PCR and Western blot after miR-636 mimic and oe-Gli2 were transfected into cancer cells; **c** Cell viability, **d** migration, **e** cell cycle and **f** the expression of Hh as well as EMT markers were detected by MTT assay, wound healing assay, flow cytometry and Western blot in each treatment group; *p < 0.05, **p < 0.01, ***p < 0.001

To further study the mechanism by which miR-636 targets Gli2 to regulate cell proliferation and EMT, oe-Gli2 and miR-636 mimic were co-transfected into HO-8910PM cells. qRT-PCR and Western blot showed that the expression of Gli2 in miR-636 mimic + oe-Gli2 group was similar to that in mimic-NC + oe-NC group (Fig. 5a, b). MTT assay and wound healing assay suggested that suppression of cell proliferation and migration induced by miR-636 overexpression was reversed when Gli2 and miR-636 were simultaneously overexpressed (Fig. 5c, d). Flow cytometry was performed to evaluate the change of HO-8910PM cell cycle after Gli2 and miR-636 were simultaneously overexpressed, finding that the blocking effect of miR-636 on the cell cycle was attenuated by overexpressing Gli2 (Fig. 5e). Additionally, expression of the proteins associated with the Hh signaling pathway (Smo, Gli2) and EMT (Vimentin, E-cadherin) was measured, showing that overexpressed Gli2 reversed the inhibitory effect of miR-636 on the Hh signaling pathway and promoted the EMT process of HO-8910PM cells (Fig. 5f). Collectively, these findings illustrated that miR-636 inhibited cell proliferation, migration, blocked cell cycle and suppressed the Hh signaling pathway and EMT process in OVC by targeting to inhibit Gli2 expression, while the such effects could be reversed by overexpressed Gli2.

### In vivo experiments prove that miR-636 inhibits EMT and occurrence of OVC in mice by targeting Gli2

**Fig. 6:**
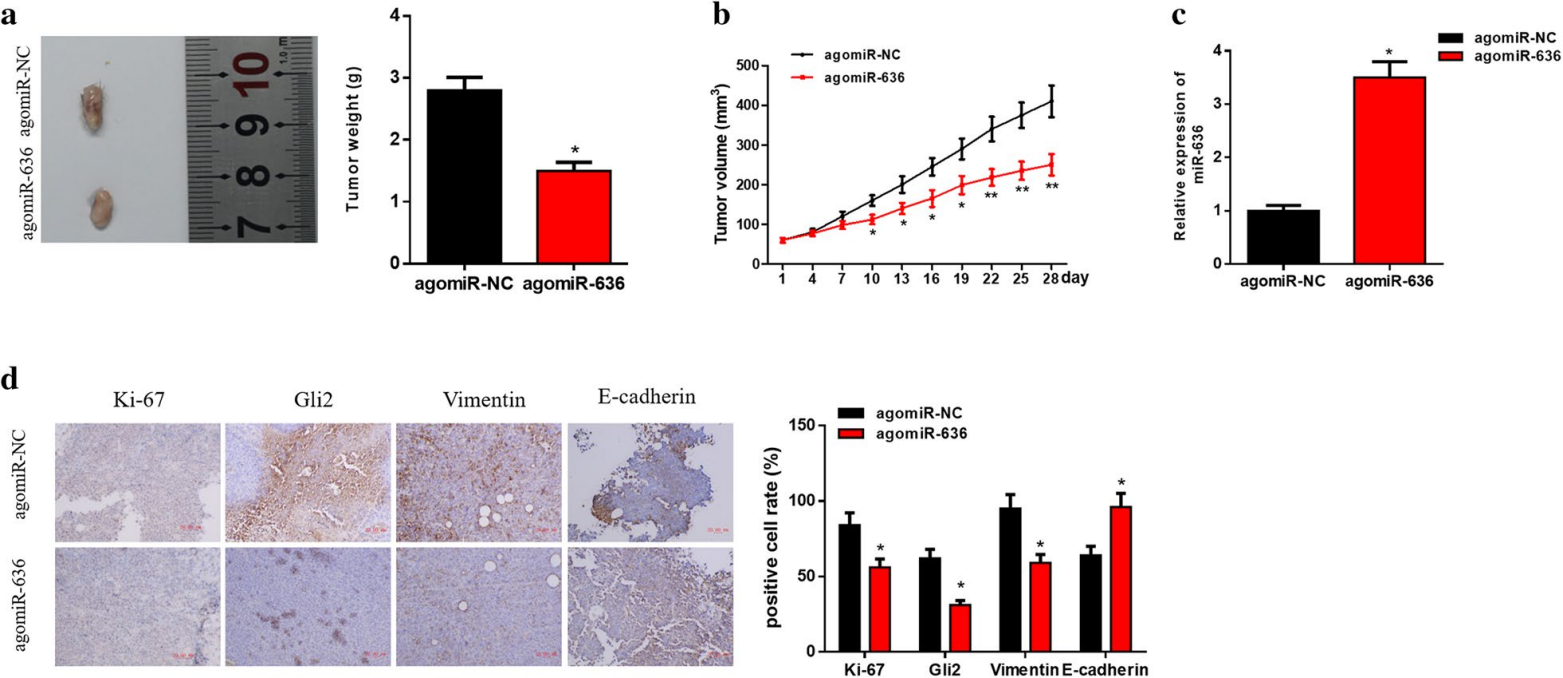
Xenotransplantation experiment proves that miR-636 inhibits the EMT and development of OVC by targeting Gli2. **a** Tumor growth and **b** tumor volume of mice after injection of agomiR-636; **c** Relative expression of miR-636 in each treatment group was detected by qRT-PCR; **d** Expression of Gli2, Ki-67, Vimentin and E-cadherin in mice tumors were examined by IHC; *p < 0.05

 To explore the effect of miR-636 on OVC occurrence in nude mice, HO-8910PM cells were firstly inoculated into mice to construct xenograft tumor models. Subsequently, agomiR-636 or agomiR-NC was injected into the mice tumors twice a week for consecutive 4 weeks. It could be seen from Fig. 6a, b that agomiR-636 significantly retarded the growth of OVC tumor in mouse models. Additionally, qRT-PCR was employed to detect miR-636 expression in the tumors, finding that miR-636 was markedly up-regulated in the agomiR-636 group relative to that in the agomiR-NC group (Fig. 6c). Moreover, the results of IHC revealed that Ki-67, Gli2 and Vimentin were obviously down-regulated in the agomiR-636 group, while the expression level of E-cadherin was remarkably up-regulated (Fig. 6d). Taken together, these findings validated that miR-636 inhibited the EMT and growth of OVC tumor in mice by silencing Gli2, and injection of agomiR-636 could effectively suppress tumor growth in the xenograft models.

## Discussion

With the development of sequencing technology, numerous miRNAs have been discovered and reported to play a critical role in occurrence and development of various cancers. Besides, miRNAs can act as a biomarker or a therapeutic target to bring novel treatment strategies for cancers [28, 29]. miR-636 is a type of non-coding small RNA that locates on chromosome 17q25.1. A recent study indicated that miR-636 suppressed cell survival of cervical cancer by targeting CDK6/Bcl-2 [30]. While in other studies, miR-636 was found to be one of the biomarkers in patients with prostate cancer and urothelial carcinoma, and it could be applied in early diagnosis of patients with prostate cancer [31, 32]. Nevertheless, studies on the role of miR-636 in OVC remain scarce. In this study, since miR-636 was found to be highly down-regulated in OVC cells, we speculated that miR-636 may function as a tumor suppressor in OVC. Additionally, we conducted bioinformatics analysis and discovered that miR-636 could target to Gli2 in the Hh signaling pathway, thereby reducing the activation of the Hh signaling pathway and suppressing the EMT in OVC. The Hh signaling pathway is highly conserved in evolution in insects and mammals and plays an essential role in embryonic development and morphogenesis [33]. In mammals, Hh signal transduction happens primarily in cilia structure of cells. Inadequate Hh signals can result in congenital developmental abnormalities (cyclops, holoprosencephaly, etc.), while excessive Hh signals can lead to basal cell carcinoma of the skin, medulloblastoma and other tumors [11, 34]. Gli2 is one of zinc finger transcription factors in the Hh signaling pathway. Several studies found that abnormal activation of Gli2 results in the occurrence of various tumors, such as colorectal cancer, glioma, cervical cancer and bladder cance r[35–38]. Similarly, there are also multiple literatures indicating that Gli2 is abnormally highly expressed in various tumors, which helps the development of EMT process [39–41].

Here, we found that miR-636 was highly down-regulated in OVC tissue and cells, and was significantly associated with the expression of the Hh signaling pathway and EMT markers. It was also revealed that overexpressing miR-636 markedly suppressed OVC cell proliferation and migration, and induced cell cycle arrest in G0/G1 phase. While miR-636 was silenced, significantly promoted cell proliferation and migration were noted, as well as increased Vimentin and decreased E-cadherin, which indicates the promotion of the EMT process. In the meantime, xenotransplantation experiments validated that miR-636 inhibited tumor growth and EMT of OVC in vivo. For discussion of downstream regulatory mechanism, dual-luciferase assay identified that miR-636 could directly target Gli2 in OVC cells. In OVC cells, overexpressing miR-636 decreased Gli2 expression, while Gli2 expression was increased after miR-636 was knocked down. In order to further investigate the effect of Gli2 on the occurrence and development of OVC, Gli2 was overexpressed or silenced in OVC cells. Results revealed that overexpression of Gli2 remarkably promoted cell proliferation and migration, which is in consistent with the results when miR-636 was inhibited. While Gli2 was silenced, it was found to significantly inhibit cell proliferation and migration and block cell cycle in G0/G1 phase, which is also in line with the results upon overexpression of miR-636. Furthermore, rescue experiments were carried out and it was revealed that the regulatory effect of miR-636 on OVC cells could be partially reversed by Gli2 after miR-636 and Gli2 were simultaneously overexpressed. Collectively, our findings illustrated that miR-636 could inhibit the EMT, cell proliferation and cell cycle by directly targeting the transcription factor Gli2 of the Hh pathway in OVC.

## Conclusions

**Fig. 7:**
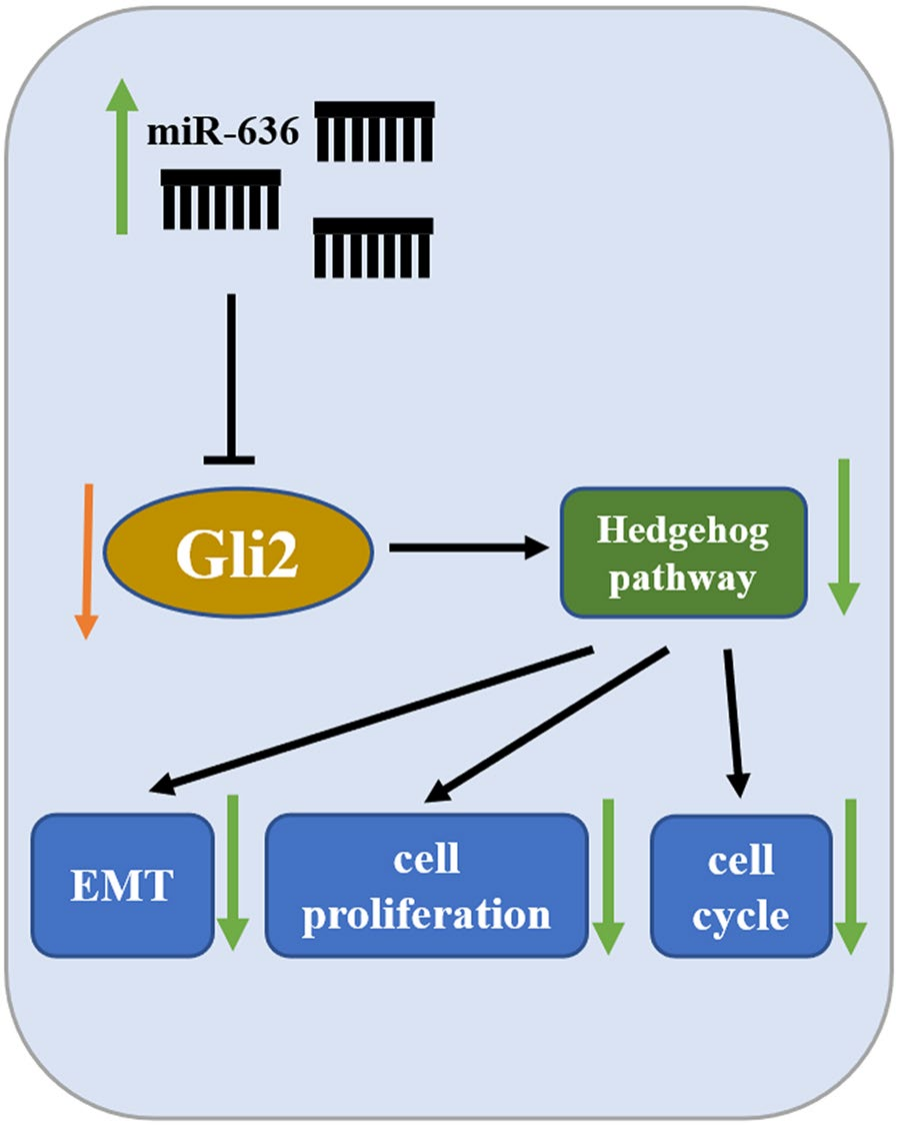
Schematic diagram of proposed mechanism. miR-636 may target the transcription factor Gli2 of the Hh signaling pathway to suppress the EMT, cell proliferation and cell cycle of OVC

In summary, this study found that miR-636 was lowly expressed in OVC, and overexpressing miR-636 inhibited cell proliferation and migration as well as blocked cell cycle in G0/G1 phase. Additionally, we also discovered that miR-636 regulated the Hh signaling pathway by targeting Gli2, and in turn suppressing the cell proliferation, migration, inducing the cell cycle arrest and impeding the EMT process (Fig. 7). Our achievements not only allow us to gain more insight into the role of miR-636 in OVC, but also lay a foundation for searching new approaches on targeted therapy for OVC. Indeed, there are some limitations that are still present in this research. For instance, the association of miR-636/Gli2 with clinical pathological characteristics and prognosis of OVC patients is not involved. Given this, we will collect more clinical samples and schedule a follow-up visit to gather more information for further analysis. Besides, this study is limited to only three aspects, including EMT, cell proliferation and cell cycle. Since cells are complicated, we will move on to study the effect of miR-636/Gli2 on other cell biological processes.

## Supplementary Information

**Additional file 1:** **Table S1.** All primer sequences used in experiments. **Table S2.** Information for all antibodies used in experiments. **Table S3.** Hh signaling pathway related genes

## Data Availability

The data and materials in the current study are available from the corresponding author on reasonable request.
