# Supplementary material for: miR-636 inhibits EMT, cell proliferation and cell cycle of ovarian cancer by directly targeting transcription factor Gli2 involved in Hedgehog pathway
Source: Cancer Cell Int. 2021 Jan 20;21:64. doi: 10.1186/s12935-020-01725-7 (PMC7819188; doi:10.1186/s12935-020-01725-7)
Supplement: Supplementary file 1 — Additional file 1: Table S1. All primer sequences used in experiments. Table S2. Information for all antibodies used in experiments. Table S3. Hh signaling pathway related genes [file 12935_2020_1725_MOESM1_ESM.docx]

**Supplementary Table 1 All primer sequences used in experiments**

| **Gene** | **Sense sequence** **(5’-3’)** | **Anti-sense sequence (5’-3’)** | **Amplicon size** |
| --- | --- | --- | --- |
| miR-636  NR_032659.1 | TCGGCAGGTCGATACGGTCGTA | CTCAACTGGTGTCGTGGA |  |
| Smo  NM_001105565.3 | GCTGGGTCCAGACTGTGTAG | GCTGCCTTAGTGTTGCGGA | 249 |
| Snail  NM_178310.4 | ACTGCGACAAGGAGTACACC | GAGTGCGTTTGCAGATGGG | 220 |
| Tgfβ1  NM_015927.5 | TACAGCACGGTATGCAAGCC | GCAACCGATCTAGCTCACAGAG | 112 |
| E-cadherin  NM_001317185.2 | AGGCCAAGCAGCAGTACATT | ATTCACATCCAGCACATCCA | 110 |
| Vimentin  NM_001017921.4 | GCCCTAGACGAACTGGGTC | GGCTGCAACTGCCTAATGAG | 137 |
| Gli2  NM_001374353.1 | \| CTGCCTCCGAGAAGCAAGAAG \| \| --- \| | GCATGGAATGGTGGCAAGAG | 157 |
| U6[42] | CTCGCTTCGGCAGCACA | AACGCTTCACGAATTTGCGT |  |
| GAPDH  NM_002046 | ACAACTTTGGTATCGTGGAAGG | GCCATCACGCCACAGTTTC | 101 |

**Supplementary Table 2 Information for all antibodies used in experiments**

| **Antibody** | **WB/ (IHC)** | **Specificity** | **Company** |
| --- | --- | --- | --- |
| GAPDH | 1/10000 | Rabbit monoclonal | Abcam, China |
| E-cadherin | 1/10000 (1/500) | Rabbit monoclonal | Abcam, China |
| Smo | 1:10000 | Rabbit monoclonal | Abcam, China |
| Gli2 | 1 µg/ml (1/200) | Rabbit polyclonal | Abcam, China |
| Vimentin | 1/1000 (1/200) | Rabbit monoclonal | Abcam, China |
| Ki-67 | 1/5000 (1/500) | Rabbit monoclonal | Abcam, China |
| IgG H&L (HRP) | 1:3000 | Rabbit monoclonal | Abcam, China |

**Supplementary Table 3 Hh signaling pathway related genes**

|  | **Gene name** |
| --- | --- |
| K02161c | BCL2 |
| K03083 | GSK3B |
| K03347 | CUL1 |
| K03362 | FBXW1_11 |
| K03869 | CUL3 |
| K04345 | PKA |
| K04439 | ARRB |
| K04503 | CCND1 |
| K04678 | SMURF |
| K06225 | PTCH1 |
| K06226 | SMO |
| K06229 | SUFU |
| K06230 | GLI3 |
| K06231 | HHIP |
| K06232 | GAS1 |
| K06233 | LRP2 |
| K08439 | GPR161 |
| K08957 | CSNK1A |
| K08958 | CSNK1G |
| K08959 | CSNK1D |
| K08960 | CSNK1E |
| K10151 | CCND2 |
| K10394 | KIF3A |
| K10523 | SPOP |
| K10604 | MGRN1 |
| K11101 | PTCH2 |
| K11988 | SHH |
| K11989 | IHH |
| K11990 | DHH |
| K16797 | GLI1 |
| K16798 | GLI2 |
| K18806 | KIF7 |
| K19605 | EVC1 |
| K19608 | EVC2 |
| K20020 | BOC |
| K20033 | CDON |
| K23663 | MOSMO |
| K23664 | MEGF8 |
